# Immunological consequences of compromised ocular immune privilege accelerate retinal degeneration in retinitis pigmentosa

**DOI:** 10.1186/s13023-022-02528-x

**Published:** 2022-10-17

**Authors:** K. Varsha Mohan, Alaknanda Mishra, Abaranjitha Muniyasamy, Prakriti Sinha, Parul Sahu, Ashwani Kesarwani, Kshama Jain, Perumal Nagarajan, Vinod Scaria, Manisha Agarwal, Naseem S. Akhter, Chanda Gupta, Pramod Upadhyay

**Affiliations:** 1 National Institute of Immunology, Aruna Asaf Ali Marg, New Delhi, 110067 India; 2 CSIR Institute of Genomics and Integrative Biology, South Campus, Mathura Road, New Delhi, 110025 India; 3 Dr Shroff’s Charity Eye Hospital, Kedarnath Lane, Daryaganj, New Delhi, Delhi 110002 India

**Keywords:** Retinitis pigmentosa, Tight junction protein (TJP), Blood brain barrier (BRB), Immune infiltration, Transforming growth factor—beta 1 (TGF-B1), Retinal Degeneration 1 (rd1)

## Abstract

**Background:**

Retinitis pigmentosa (RP) is a hereditary retinal disease which leads to visual impairment. The onset and progression of RP has physiological consequences that affects the ocular environment. Some of the key non-genetic factors which hasten the retinal degeneration in RP include oxidative stress, hypoxia and ocular inflammation. In this study, we investigated the status of the ocular immune privilege during retinal degeneration and the effect of ocular immune changes on the peripheral immune system in RP. We assessed the peripheral blood mononuclear cell stimulation by retinal antigens and their immune response status in RP patients. Subsequently, we examined alterations in ocular immune privilege machineries which may contribute to ocular inflammation and disease progression in rd1 mouse model.

**Results:**

In RP patients, we observed a suppressed anti-inflammatory response to self-retinal antigens, thereby indicating a deviated response to self-antigens. The ocular milieu in rd1 mouse model indicated a significant decrease in immune suppressive ligands and cytokine TGF-B1, and higher pro-inflammatory ocular protein levels. Further, blood–retinal-barrier breakdown due to decrease in the expression of tight junction proteins was observed. The retinal breach potentiated pro-inflammatory peripheral immune activation against retinal antigens and caused infiltration of the peripheral immune cells into the ocular tissue.

**Conclusions:**

Our studies with RP patients and rd1 mouse model suggest that immunological consequences in RP is a contributing factor in the progression of retinal degeneration. The ocular inflammation in the RP alters the ocular immune privilege mechanisms and peripheral immune response. These aberrations in turn create an auto-reactive immune environment and accelerate retinal degeneration.

**Supplementary Information:**

The online version contains supplementary material available at 10.1186/s13023-022-02528-x.

## Background

Retinitis Pigmentosa (RP) is a hereditary ocular disease which causes photoreceptor loss [1] due to mutations found in over 100 genes [2, 3]. Despite the shared consequence of vision loss among the mutations, non-genetic components [4] such as ocular inflammation can contribute to retinal degeneration [5–7].

Studies indicate that the damaged photoreceptor releases pro-inflammatory molecules resulting inflammation [8] in the eye. The intra-ocular inflammation in several ocular diseases compromised the ocular immune privilege mechanisms [9]. Further, it has been shown that the sustained chronic inflammatory reaction may contribute to the pathogenesis of retinal degeneration in mice [7]. Similarly, the cytokine profile in the RP patient suggests the sustained chronic inflammatory reaction may underlie the pathogenesis in RP [5, 6] and ocular inflammation [10–13]. Several studies have reported retinal leakage and antibodies against ocular antigens in RP [12, 14–18] due to inflammatory changes in the eye.

However, the consequences of ocular inflammation on ocular immune privilege which can exacerbate the disease remains to be explored. The ocular immune privilege protects the eye and it excludes peripheral immune response by mechanisms such as the physical barrier, the antigen exclusion, the immune suppressive molecules and its anti-inflammatory milieu [19]. Further, Anterior chamber associated immune deviation (ACAID) prevents peripheral immune activation against antigen introduced in the aqueous humour [8, 19].

Since the inflammatory changes in the eye can compromise the ocular immune deviation, we aimed to study the status of the ocular immune privilege during retinal degeneration and the effect of ocular immune changes on the peripheral immune system in RP.

We investigated systemic immune cell activation upon self-retinal antigen stimulation in peripheral blood mononuclear cells (PBMCs) derived from RP patients followed by a study into the ocular immune changes in rd1 mouse model. Our study attempts to explain how the breakdown of ocular immune privilege mechanisms can affect the rate of retinal degeneration in RP.

## Results

### RP patient peripheral blood mononuclear cell stimulation

**Fig. 1:**
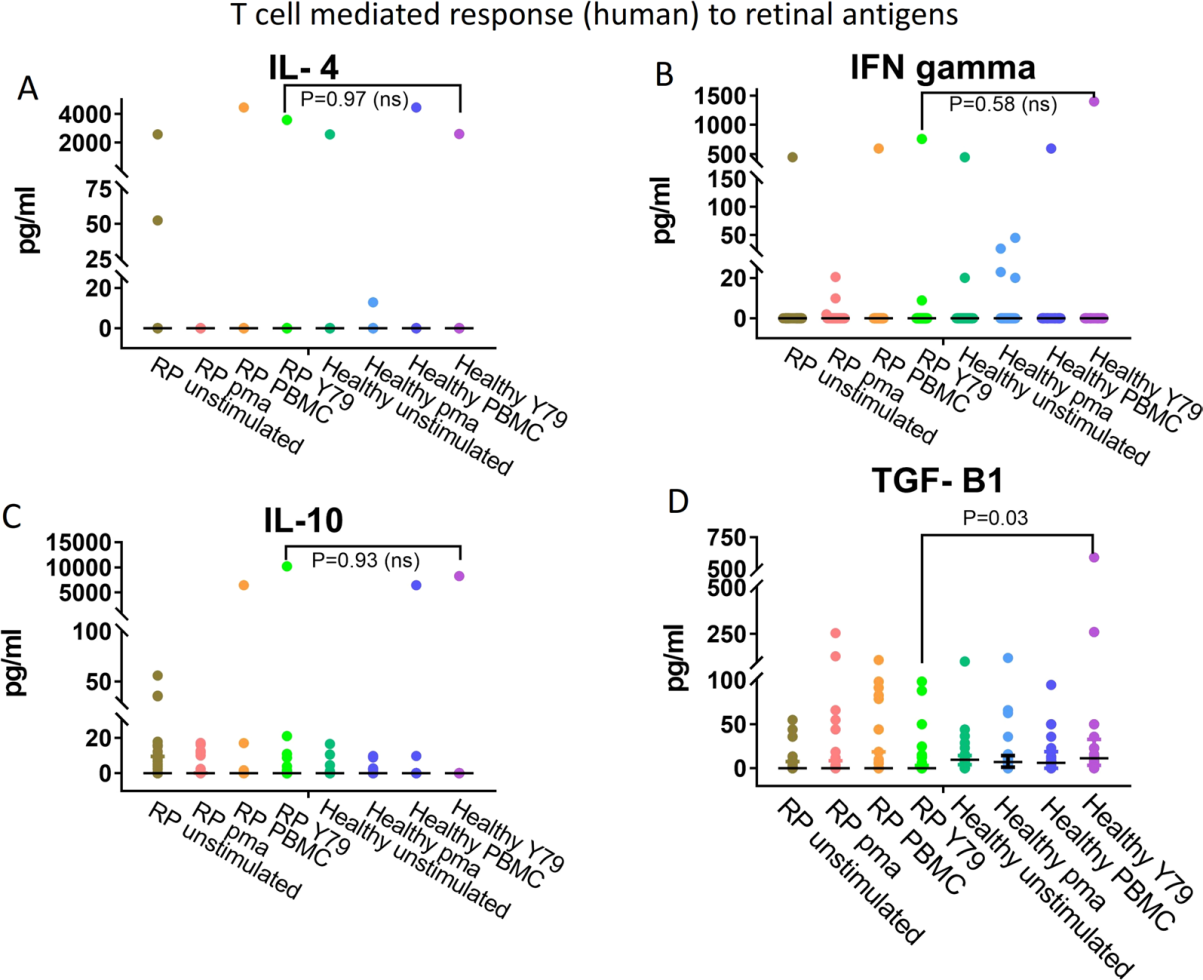
T cell mediated response to retinal antigens. Cytokine secretion profiles of mononuclear cells upon stimulation. Classically anti-inflammatory response is elicited against self-antigens, however RP patients displayed significantly lower anti-inflammatory TGF-B1 (**D**) and slightly reduced IL10 (**C**) cytokine response to retinal antigens. While IL4 (**A**), IFNg (**B**) levels were non-significant. RP n = 40, healthy n = 30

The Y79 retinoblastoma cell line lysate was used to investigate the peripheral T cell-mediated response to retinal antigens in RP patients. The PBMCs isolated from RP patients were stimulated with Y79 cellular lysate and IL4, IFNg, IL10 and TGF-B1 levels was measured. PBMC isolated from RP patients displayed significantly lower TGF-Β1 (*P* = 0.03) response than the control (Fig. 1D). However, for IL4 and IFNg the differences in response were non-significant. The cytokine secretion from unstimulated group was used as baseline secretion. The PMA stimulation activated all immune response by PBMCs which was higher than the unstimulated group. Stimulation with self-antigen using PBMC lysate showed very low or no pro-inflammatory immune response, though slightly higher TGF-B1 secretion was detected (*p* = 0.03) indicating self-antigen restriction by the T Cells in RP. This suggests that due to retinal degeneration in RP, the systemic immune system displayed reduced expression of anti-inflammatory cytokines.

### Ocular environment of retinitis pigmentosa rd1 model

**Fig. 2:**
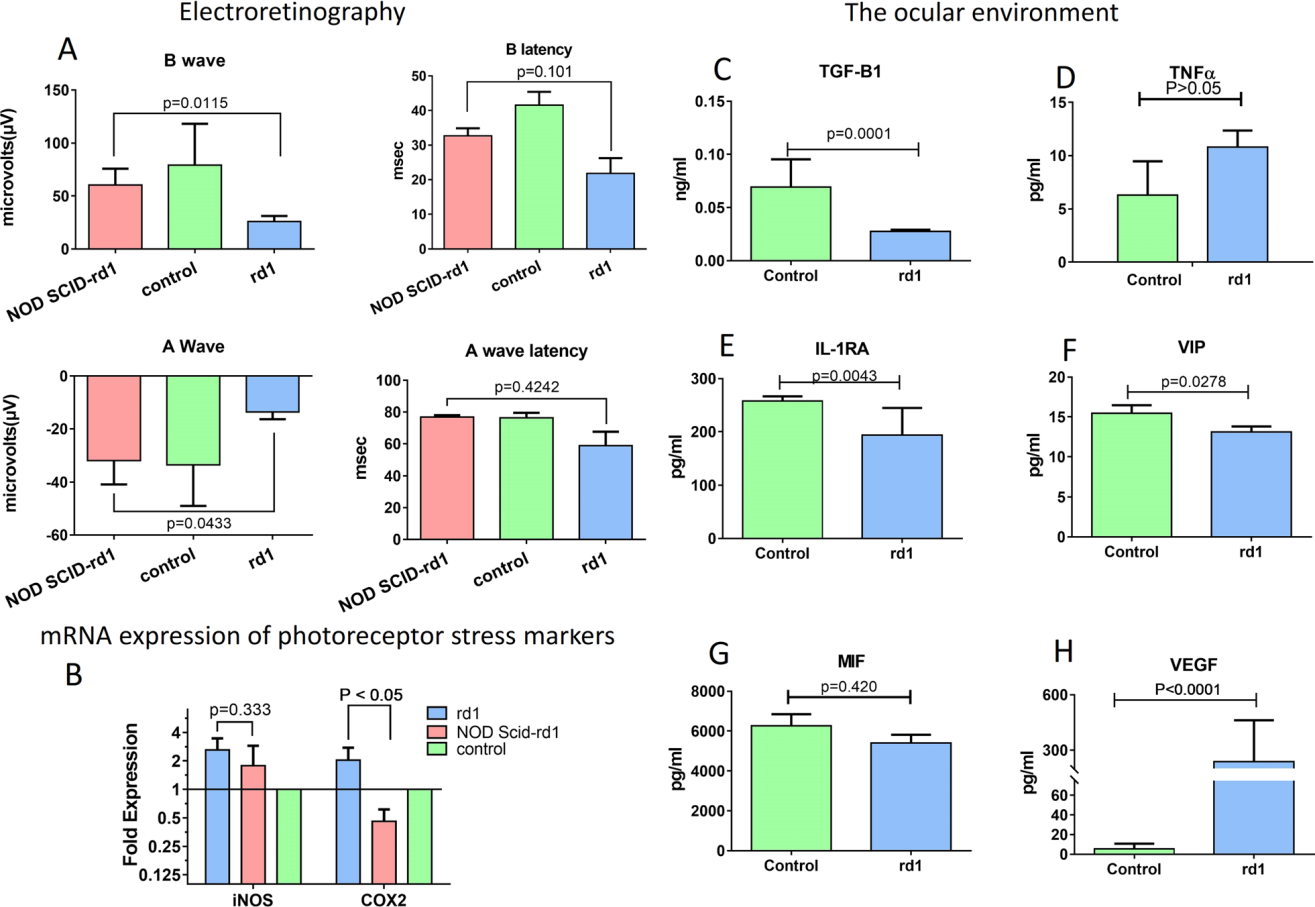
Retinitis pigmentosa animal models. Electroretinography was performed on immune competent rd1, NOD SCID-rd1 and control C57BL/6. A wave indicates the photoreceptor function while B wave indicates secondary retinal cell function responsible for signal transduction. In **A**, A wave functions displayed significant trends of vision conservation in NOD SCID-rd1 and the B wave function was conserved in NOD SCID-rd1 against rd1 and control at 4 weeks. The mRNA expression profile photoreceptor stress markers, iNOS and COX2, indicated that rd1 retina experienced higher levels of trauma in rd1 due to stress as compared to NOD SCID-rd1 and healthy retinas (**B**). The ocular environment of rd1 and control was studied which indicated that ocular environment can become pro-inflammatory due to TGF-B1 impairment as the TGF-B1 was significantly low in rd1 (**C**) while inflammatory cytokine TNFα level was increased in rd1 ocular milieu (**D**). Immune suppressive intra-ocular molecules IL-1RA (**E**) and VIP (**F**) and MIF levels (**G**) were decreased in rd1 ocular environment. This can potentially cause immune active T cells and monocytes to elicit potent responses. Elevated level of intra-ocular VEGF was detected in rd1 (**H**) which is an indication of ocular inflammation and pathogenic angiogenisis. n = 5

Full field electroretinography was performed on control C57BL/6 J, NOD SCID-rd1 and CBA/J mice (n = 5). A and B wave functions represent the photoreceptor and secondary signalling retinal cells functions respectively. A wave function displayed trends of vision conservation in NOD SCID-rd1. The A wave and B wave functions were significantly different between rd1 and NOD SCID-rd1 (*p* = 0.0433 and *p* = 0.0115 respectively) (Fig. 2A). The mRNA expression profile of photoreceptor stress markers iNOS and COX2 were also evaluated in control C57BL/6, NOD SCID-rd1 and CBA/J rd1 (n = 9), wherein rd1 expressed higher levels of stress marker iNOS and significantly higher COX2 (*p* < 0.05) than adaptive immune-compromised NOD SCID-rd1 (Fig. 2B). The anti-inflammatory nature of ocular milieu was assessed in rd1 and control (n = 5) and significantly reduced levels of ocular TGF-Β1 (*p* < 0.0001) (Fig. 2C). Flow cytometry studies also confirmed lower level of TGF-B1 in the rd1 eye compared to the C57BL/B6, plots are shown in Additional file 1: Fig. S2, panel A.

The pro-inflammatory TNFα level (Fig. 2D) was observed in rd1 ocular milieu. Further, ocular immunosuppressive molecules and VEGF were evaluated wherein MIF (Fig. 2G), IL1RA (*p* = 0.0043) (Fig. 2E), VIP (*p* = 0.0278) (Fig. 2F) were significantly downregulated while VEGF (*P* < 0.0001) (Fig. 2H) was significantly over-expressed in rd1. Similar trend was observed in western blot of VEGF, where it was overexpressed in the rd1 eye compared to the control C57BL/B6 as given Additional file 1: Fig. S2, panel B and C.

### Blood–retinal-barrier

**Fig. 3:**
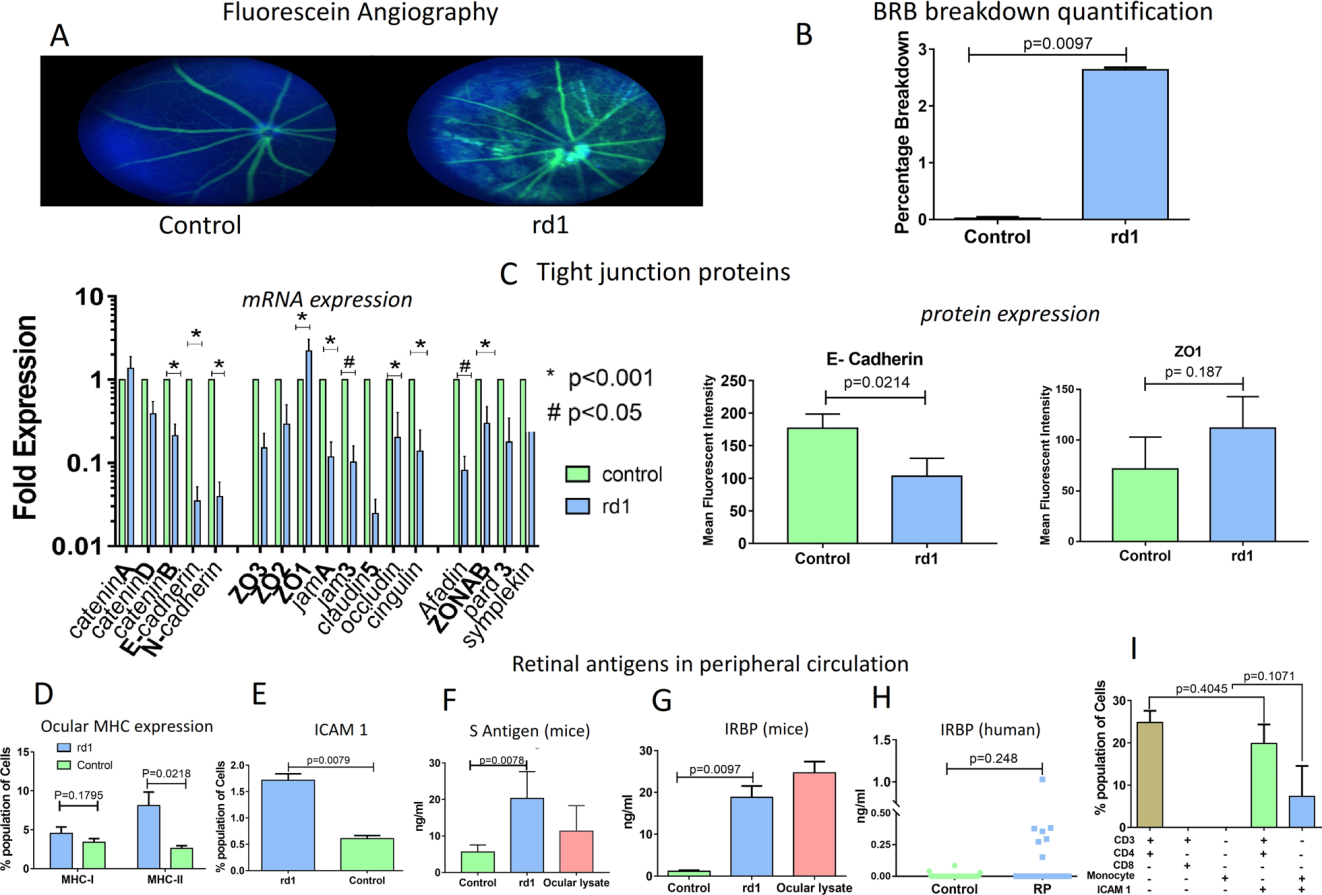
Blood-retinal-Barrier Study. Sodium angiography of control and rd1 indicated that the Blood retinal barrier is compromised, as seen by the extravasation dye into retina from retinal vessels (**A**, **B**). The mRNA expression profile of ocular tight junction protein displays a significant down-regulation of all proteins except ZO1 in rd1. ZO1, a RPE replication inhibiting factor is significantly up-regulated, the expressed proteins follow similar trend (**C**). The E cadherin was found to be significantly downregulated in rd1 (*p* = 0.0214) and ZO1 was overexpressed in rd1 RPE cells compared to C57BL/B6 (n = 3). The rd1 RPE layer displayed significantly higher number MHC-II expressing RPE cells which otherwise expresses only MHC-I (**D**). ICAM1 adhesion protein expressing RPE cells, responsible for immune cell extravasation, were also significantly increased in rd1 (**E**). Retinal S antigen (**F**) and IRBP (**G**) were significantly higher in rd1 peripheral circulation as compared to control. Ocular lysate was used as positive control for the retinal antigens. IRBP antigen level in serum was higher in RP patients (n = 40) compared to healthy group (n = 30) (**H**). Suppression of ICAM1 by neutralizing antibodies can deter monocytes. However, T-helper CD3+4+ cells can infiltrate even in the absence of ICAM1 adhesion ligand on RPE (**I**)

The integrity of the BRB was assessed by fluorescein angiography, wherein the control eye demarcated the retinal vessels and showed an absence of extravasation of the dye, while the extravasation of fluorescein dye in the rd1 retina indicated a BRB breakdown (Fig. 3A). A typical angiography image of NOD SCID-rd1 is given in Additional file 1: Fig. S3. The ocular fluorescein level was quantified to evaluate the breakdown percentage which indicated a significant (*p* = 0.0097) 2.8% breakdown in rd1 (n = 5) (Fig. 3B). The mRNA profile of rd1 (n = 9) indicated an overall significant downregulation of the BRB tight junction protein (*p* < 0.001) and adhesion proteins (*p* < 0.05) except the ZO1 (*p* < 0.01) which was upregulated (Fig. 3C). The expressed proteins follow similar trend; E cadherin was significantly downregulated (*p* = 0.0214) and ZO1 was overexpressed in the rd1 (Fig. 3C and Additional file 1: Fig. S5). This was reconfirmed by the fluorescence microscopy images showing identical trend (Additional file 1: Fig. S4).

Further, the flow cytometry study of antigen presenting and immune cell extravasation ligand indicated a decrease in number of MHC-I expressing RPE cells, while a significant increase in MHC-II (*p* = 0.0218) and ICAM1 (*p* = 0.0079) expressing retinal pigment epithelium cells in rd1 than control (n = 5) (Fig. 3D, E). The level of retinal antigens in peripheral circulation as a consequence of BRB breakdown was evaluated, and a significant increase in retinal S antigen (*p* = 0.0078) (Fig. 3F) and IRBP (*p* = 0.0097) (Fig. 3G) was observed in the rd1 serum compared to control (n = 5). IRBP retinal antigen was also detected in the peripheral circulation of RP patients (Fig. 3H) ICAM1 neutralization assay indicated a decrease in monocyte extravasation into rd1 eye however T cells showed no deterrence in infiltration on masking the ICAM1 (Fig. 3I).

### Ocular–systemic interaction

**Fig. 4:**
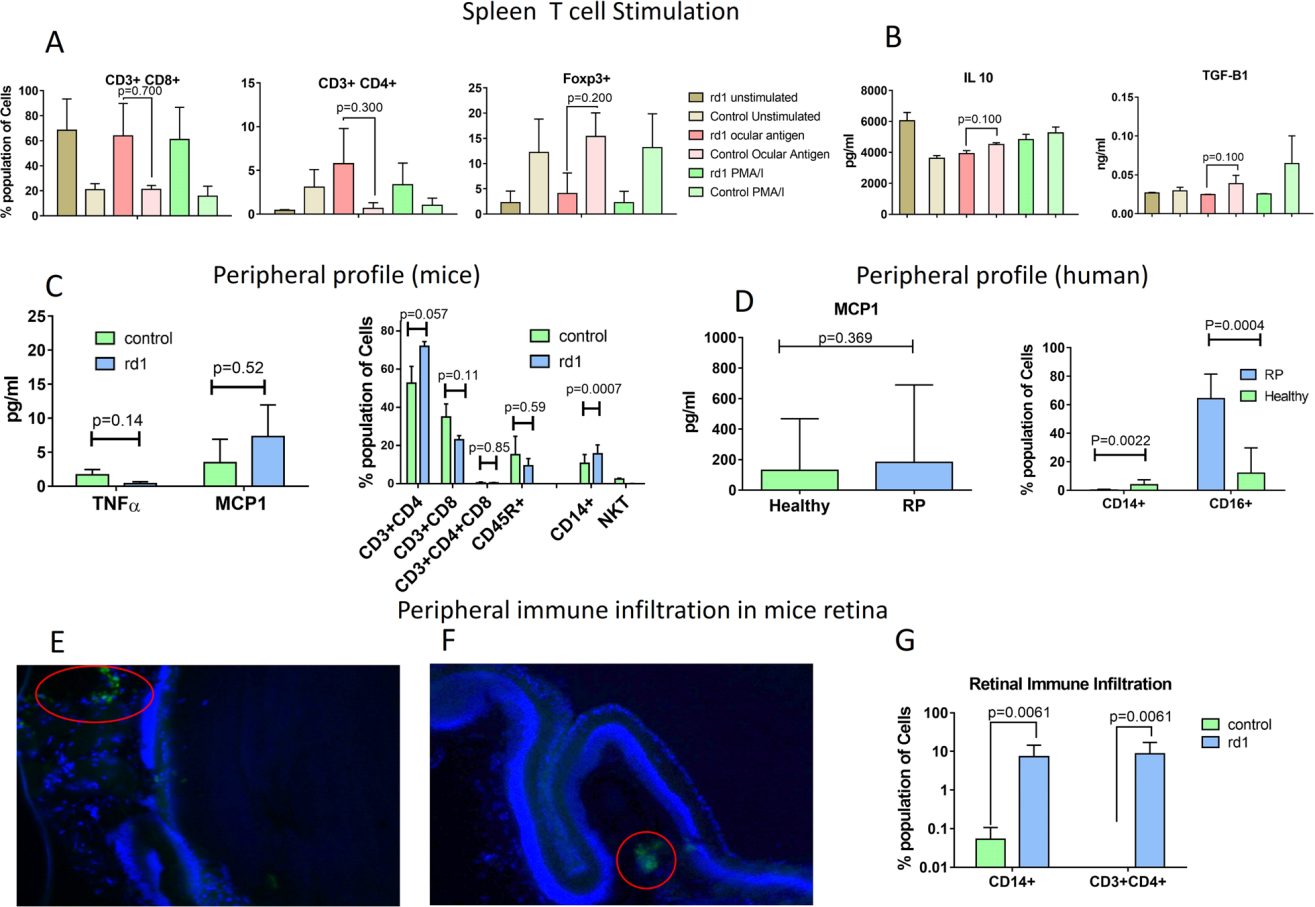
Ocular-systemic interaction study. T cells in the spleen mediate a tolerogenic response to retinal antigens by IL10 and TGF-B1 secreting Tregs. Spleen mononuclear cells stimulated with self-retinal antigens displayed higher CD4 + Th response in rd1 while control displayed higher Foxp3 + Treg response (**A**). The stimulated mononuclear anti-inflammatory cytokine profile of rd1 displayed lower secretion of IL10 and TGF-B11 than control (**B**). MCP1 secretion was higher in rd1 peripheral circulation despite lower TNFα levels, indicating monocytic activation in absence of systemic inflammation (**C**). CD4+ Th cells and monocytes levels were higher in the peripheral system of rd1 indicating cellular specific activation (**C**). Slightly elevated levels of MCP1 and significantly higher number of monocytes of M2 origin was observed in RP patients’ peripheral circulation (**D**). Monocytes (**E**) and T cells (**F**) infiltrate the immune privileged retina of rd1 as seen by IHC and flow cytometry study of infiltrating peripheral immune cells in rd1 retina (**G**)

The spleen mononuclear cells stimulation with retinal antigens in ocular lysate was evaluated to examine the T cell response as a part of peripheral immune response. The rd1 (n = 3) displayed a higher CD3+ CD4+ cells to retinal antigens while the control displayed higher Foxp3+ T cells (Fig. 4A). The rd1 also showed lower IL10 and TGF-Β1 secretion upon retinal antigenic stimulation (Fig. 4B). Peripheral blood profiling in rd1 indicates an increase in the MCP1 levels in the rd1 serum. Additionally, an increase in CD3+ CD4+ T cells and significant increase in monocytes (*p* = 0.0007) was observed in rd1 peripheral blood profile (n = 5) (Fig. 4C).

Similar peripheral blood profile was observed in RP patients, CD45+ CD16+ non-classical monocytes were significantly (*p* = 0.0004) higher than control (RP = 40, Control = 30) and classical CD45+ CD14 monocytes were also significantly elevated (*p* = 0.0022). However, there was a lot of variations in MCP1 levels within RP and healthy samples and the overall difference between the two was statistically non-significant (*p* = 0.369) (Fig. 4D). Flow cytometry study of rd1 ocular immune cells infiltration indicated the significant presence of CD3 + 4 + T cells and monocytes (*p* = 0.0061) (Fig. 4G). ICC studies showed that the T cells and monocytes could cross the BRB and infiltrate the retina (Fig. 4E, F).

## Discussion

The role of inflammation and its subsequent consequences on ocular immune privilege in genetic diseases such as RP is not well understood. Several isolated evidences do indicate immune involvement in the pathogenesis [20, 21], and these can contribute to exacerbate the disease [22]. Recent studies by Kyger et al., has shown the presence of pathogenic auto-antibodies and T cell activation against retinal antigens in Royal College of Surgeons (RCS) rats [23]. Further, the presence of retinal auto-antibodies in RP patient’s blood has been reported [24]. Since the generation of IgG [25] producing B cells requires antigen specific T cell activation [26], the nature T cell response is of keen interest.

We began the study by assessing the peripheral blood mononuclear cells (PBMCs) response upon retinal antigenic stimulation to gauge T cell response in RP patients. The immune tolerance against few retinal antigens is maintained by eliminating reactive T cells during thymic selection and ocular immune-suppressive molecules turns auto-reactive T cell to Tregs [27]. Further, peripheral immune tolerance mediates Tregs to elicit anti-inflammatory immune response mediated by TGF-Β1 and IL10 against retinal antigens, thereby neutralizing pathogenic immune response against auto-antigens [28]. However, RP patients displayed significantly lower TGF-Β1 and lower IL10 response than control. The decreased peripheral anti-inflammatory response to retinal antigens could be a possible consequence of compromised ocular immune deviation system [19]. The IL4 and IFNg response were non-significant as T cells response to non-retinal epitopes in Y79 lysate which can mask any IL4, IFNG mediated T cells response estimation against retinal antigens. Since our interest was to study the nature anti-inflammatory response mediated by IL10 and TGF B1 against retinal antigens such masking is unlikely to affect the preliminary result.

The investigations on changes that lead to the ocular privilege breakdown were carried out in RP mouse model rd1 and its immune-compromised NOD SCID-rd1 variant [29]. The rd1 is a rapid progressing autosomal recessive model of RP with mutation in Pde6b. The rate of progression causes the rd1 model to be devoid of photoreceptors by 4 weeks. Other commonly used RP model includes rd10. Rd10 mutation is a slow progressing RP model with missense mutation in exon 3 in Pde6bn and is devoid of photoreceptors by 3 months. Rd10 has been commonly used due to the slow progression which allows a window for therapeutic intervention. We used the rd1 model, which is better optimized to the time taken to manifest the severe consequence of retinal degeneration [30].

To understand whether the absence of adaptive immune system affected the pace of retinal degeneration, ERG and retinal stress markers were compared in rd1, control and NOD SCID-rd1. Electroretinographic evaluation of the retinal functions indicated significant trends of photoreceptor function conservation in the NOD SCID-rd1 mice compared to the rd1 mice. The A wave and the B wave function parameters were significantly more conserved in NOD SCID-rd1 than rd1 thereby indicating that an active immune system might be contributing to the hastening of retinal degeneration. The retinal stress markers [13, 31] indicate similar patterns wherein the NOD SCID-rd1 displayed significantly lower levels of retinal stress marker COX2 and lower iNOS than rd1. Thus, cementing the effect of antagonistic immune responses on ocular system in RP model.

Therefore, we studied the changes in the ocular immunoregulation in RP including the nature ocular milieu, immune suppressive molecules, ocular ligands, BRB and antigen exclusion from adaptive immune system.

The ocular environment is pre-dominantly anti-inflammatory and immune-suppressive [13, 32]. Any inflammatory changes in such a limited and well-defined environment will act as a trigger to potentiate further inflammatory modifications in the eye [8, 9, 13]. The study of the ocular milieu in rd1 against control indicated a significant decrease in the key resident anti-inflammatory cytokine, TGF-B1, while inflammatory TNFα levels were detected in rd1 [33]. Further VEGF[33], another pro-inflammatory ocular molecule level was significantly higher in rd1. These modulations indicate pro-inflammatory [32, 34] changes in ocular milieu in rd1.

We also observed lower levels of IL-1RA, which inactivates any infiltrating T cells, and reduced Monocyte inhibiting factor (MIF) and vasoactive intestinal peptide (VIP) levels in rd1. VIP downregulates production of IL6 and TNFα and suppresses cell mediated immunity by inhibiting the proliferation of T cells and monocytes [35, 36]. MIF contributes to immune privilege by inhibiting NK cell mediated cell lysis due to lower-than-normal MHC molecules in the eye [37]. Their decreased levels can potentially render the infiltrating peripheral immune cells functionally active.

The blood-retinal-barrier (BRB) is closely associated with the ocular milieu and the pro-inflammatory changes in the milieu would have a direct effect on it [38]. The BRB is sensitive to inflammatory changes which can cause its breakdown [38–41]. The integrity of BRB was checked using fluorescein angiography, which displayed that BRB integrity was altered in rd1. Further, the mRNA profile of BRB adhesion and tight junction proteins showed that the breakdown was systemic with a pan downregulation of the proteins [42]. However, ZO1, a tight junction protein and an inhibitor of retinal pigment epithelium cell proliferation and differentiation signalling pathway was significantly upregulated [42]. Further, protein expression profile indicated reduced E cadherin protein and higher ZO1. Thus, the breakdown is a systemic modulation of the TJPs and AdPs and not a consequence of cellular loss due to inflammatory insult [43–45]. The modulation in the TJPs and AdPs mRNA expression could be due to the TNFα signalling mediated downregulation as suggested by several studies in other ocular diseases [38, 40, 43, 44]. The downregulation of TJPs and AdPs mRNA explains the fluorescein angiography leakage at optic nerve reported by Gattegna et al. [46] and Alekseev et al. [16] in Autosomal Recessive RP (ARRP) patients. Their study indicates inflammatory feature with fluorescein angiography leakage and auto-antibodies in the RP patients, which could be a consequence of ocular inflammation and a compromised BRB.

The BRB also mediates immunomodulatory function on the peripheral circulation side of retinal pigment epithelium (RPE). RPE constantly processes the photoreceptor disc during its turnover [47] and the processed disc peptides are presented to the peripheral circulation on MHC-I as an antigen. In this process, the interacting T cells-MHC-I are turned anergic by RPE surface ligand FasL and CTLA [14, 38]. The flow cytometry investigation of cells isolated from RPE in rd1 indicated a significant increase in the number of MHC-II (and decrease in MHC-I) and ICAM1. The RPE does not conventionally express MHC-II [48] or immune cell adhesion ligand ICAM1 [13] and are expressed as a consequence of inflammatory changes in the eye.

As a result of the increased frequency of MHC-II RPE cells, they turn into a potential antigen presenting cell for retinal antigens [49], priming the reactive T cells and extravasate immune cells into the eye [48]. ICAM1 neutralization study in rd1 eye indicated that it indeed extravasates immune cells albeit only monocytes. The T cells continued to infiltrate in its absence perhaps by other ligands such as VCAM [50].

The BRB maintains antigen exclusion of ocular antigens which are normally sequestered, and prevent them from entering the peripheral circulation [41]. The rd1 displayed a significant increase in ocular antigens IRBP and S antigen in peripheral circulation, further establishing the BRB breakdown. IRBP antigen was also detected in RP patient serum indicating the failure of antigen exclusion. Large variation in levels of these antigens is most likely due to the varying severity and duration of the retinal degeneration in the patients also reported by Kumar et al. [11] among other studies [10, 18, 51].

The release of sequestered antigens into the circulation would prime the immune system as ocular immune privilege also includes ocular antigen exclusion [41, 52, 53]. Iannaccone and Radic have showed that retinal degeneration triggers retinal citrullination which can generate retinal-autoantibodies, local and systemic immune activation which in-turn contributes to disease pathogenesis [12]. Further, Epstein et al., has also indicated the presence of pathogenic anti-IRBP antibodies in TAM mice and autosomal ARAP patients [17]. However, the retinal antigens are not entirely void in healthy individuals and their non-pathogenic levels has been reported earlier [15].

Since the eye lacks lymphatic drainage, circulating antigens would reach the spleen [19]. In the spleen, the peripheral immune tolerance [54, 55] causes any T cells interacting with the ocular antigen in the peripheral circulation to elicit Treg response mediated by TGF-Β1 and IL10 [19, 22, 56]. However, in rd1 the spleen mononuclear cell stimulation with ocular antigens elicited a lower anti-inflammatory response and Tregs were lower than control. Instead, higher number of CD3+ CD4+ T cells were detected upon stimulation with retinal antigens. Similarly, in the peripheral circulation higher numbers of T cells and monocyte numbers were observed in rd1. MCP1 chemoattracts the monocytes to the site of insult and MCP1 levels were elevated in rd1. In RP patients, a higher count of CD45+ CD16+ monocytes in peripheral circulation were observed and there were large variations in MCP1 levels. These findings corroborate with studies which indicate macrophage recruitment to the damage site where further inflammation in mediated by the cells [23, 57].

The ICC and flow cytometry study of rd1 eye showed infiltration of T cells and monocytes. These cells could cross the BRB by extravasation ligand expressing on RPE and enter the retina. The absence of immune suppressive molecules allows the immune cells to effectively elicit inflammatory response against self-cells, further accelerating the retinal degeneration. Thus, the dysregulation of the several protective mechanisms compromises the ocular immune privilege and causes further damage to the eye in RP.

These findings suggest that due to the immunological consequences of retina degradation a common phenotype is manifested among RP patient’s despite of their varying genotypes, duration and severity of retinal degeneration.

## Conclusion

The eye is an extension of the brain and it is an immune-privileged organ. There are several anti-inflammatory molecules, barriers and mechanisms to suppress the immune action thus to protects the eye.

In RP, the inflammation induced by the degrading retina in the eye acts like a trigger which results in the dysregulation of the several protective mechanisms and compromises the immune privilege of the eye and causes systemic immune mediated damage. The ocular inflammation causes the suppression of anti-inflammatory molecules, cytokine and ligands.

Further, the BRB layer is sensitive to inflammatory changes. The breakdown of BRB in RP lets the leakage of sequestered ocular antigens into peripheral circulation and thereby results in a failure of antigen-exclusion. In such a situation, due to the failure of peripheral tolerance, the immune active T cells and reactive monocytes are generated which further accelerates degeneration of retina.

The ocular antigens then became capable of priming the peripheral immune cells and mediate auto-reactivity against retinal antigens in eye. The presence of surface ligands such as MHCII and ICAM1, which are typically absent on ocular cells, further leads to priming and extravasation of immune cells into an already compromised eye. Thus, the muti-fold failure of ocular immune privilege mechanisms contributes and hasten retinal loss in RP.

Our study explains that the ocular inflammation and retinal degeneration in RP exacerbate the disease due to the breakdown of ocular immune privilege mechanisms.

## Methods

### Ethics statement

This investigation was approved by the Institutional Human Ethics Committee (IHEC#100/17) of National Institute of Immunology (NII), New Delhi and the Ethic Committee, Dr Shroff’s Charity Eye Hospital (dated 22 May 2017), New Delhi. The investigation on mice was approved by the Institutional Animal Ethics Committee (IAEC# 480/18) of NII, New Delhi. All animal experiments and reporting adhere to the ARRIVE guidelines [58].

### Recruitment of RP patients

RP patients enrolled in this study were diagnosed by the retina specialist in Dr shroff eye hospital, New Delhi. The patients were genetically heterogenous and at various different stages of retina degeneration. These were diagnosed on the basis of their fundus examination, ERG and familial history. Typically, a characteristic bony spicule and retinal attenuation with the absence of anterior and posterior inflammation was seen in fundus examination of the recruited patients. Patient with autoimmune retinopathy or inflammatory ocular conditions such as uveitis was excluded. Relevant clinical details of patients are given in Additional file 1: Table ST1.

### Animal housing and breeding

CBA/J was procured from the Jackson Laboratory, USA and Nod.scid-rd1 was developed by our laboratory [29]. *rd1* is a recessive mutation model of Pde6b3′, 5′-cGMP β subunit phosphodiesterase mutation [30]. An immune compromised NOD SCID-rd1 model has been developed by our lab [29]. C57BL/6 was used as a control to study immune dysregulation in retinitis pigmentosa as some studies indicate the model to have the most stable vision perception with age and lacks spontaneous immune dysfunctions seen in other mouse models [29, 59]. All experiments were performed on 4 weeks (P28) in control, rd1 and SCID-rd1. At P28 the rd1 model is devoid of photoreceptors in the neural retina. Animals were housed in the small animal facility at National Institute of Immunology in individual ventilated cages (IVC). The animals received ad libitum access to acidified autoclaved water and food. The temperature of the housing room was maintained at 21–23 °C and animals were kept at a 14-h light-10 h dark cycle. All the experiments and procedures were performed in accordance to the guidelines issued for care and use of animals in scientific research (Indian National Science Academy, New Delhi, India).

### Blood source

In the current study peripheral blood of healthy human donors were obtained from 30 volunteers (18M/12F) at National Institute of Immunology and 40 RP patients (31M/9F) diagnosed at Dr Shroff’s Charity Eye Hospital. The age distribution was from 20 to 60 years in both the groups; for RP median age years (IQR) was 28.2 [10] and 27.4 (16.4) for healthy. Blood glucose and haemoglobin levels were assessed in both the groups to negate nutritional deficiency (Additional file 1: Fig. S1). The diagnosis of RP was based on clinical symptoms which are summarized in Additional file 1: Table ST1. RP patients were recruited randomly and not graded for the severity of disease. Volunteers with no known disease were included as controls in this study.

### PBMC isolation and stimulation

Peripheral blood mononuclear cells (PBMCs) isolated from control and patient blood (Detailed protocol is given in Additional file 1: P1 and these PBMCs were assessed for their response to retinal antigens. 10,000 cells per well was plated in a 24 well plate with RPMI and 2% antibiotic–antimycotic solution (penicillin, streptomycin and amphotericin). PBMCs were left unstimulated in 1 well and the subsequent wells were stimulated with PMA/I (500 ng/50 ng/ml; 20 μg/ml), self-PBMC lysate and retinal antigen Y79 lysate (20 μg/ml) for 24 h. The cytokine secretion in response to the stimulus was studied for the type of elicited antigenic response.

### Estimation of cytokines

Cytokine bead array (CBA) was performed for estimating IFNg, IL4, IL10, TGF-B1, TNFα and MCP1 on cell culture supernatant and mouse ocular lysate samples as per instruction manual provided by the company (BD Biosciences, USA). FCAP array software was used for the data analysis blood. Detailed protocol is given in Additional file 1: P2.

### Electroretinography (ERG)

Pupils of dark-adapted mice was dilated with 1% tropicamide and phenylephrine after dark adapting the mice for 1 h. They were then anaesthetized using ketamine (1 mg/10 g)—xylazine (0.1 mg/10 g) mixture. The gold/ active wires, ground and reference electrode were placed on the cornea, inserted in the tail and subcutaneously placed between the eyes on the forehead respectively. A white light from LED light source (intensity of 10cds/m^2^) stimulated the retina, and the consequent amplitude and latency of ‘a’ and ‘b’ wave were measured (inbuilt algorithm of LabScribe software). The mean of 25 readings was obtained for single full field ERG response and analysed (MICRON III rodent imaging system using LabScribe software, Phoenix laboratory, USA).

### Enucleation

Enucleated eyeballs were micro-dissected to obtain the posterior cup containing the neural retina layer and the retinal pigment epithelial (RPE) layer, which was used for further analysis as explained in Additional file 1: P3.

### RNA isolation and cDNA synthesis

RNA was isolated in 500 μl Trizol reagent from the homogenized posterior eye cup for gene expression profile study of control and test samples. Complimentary DNA synthesis was done with the use of iScript cDNA synthesis kit (BioRad, USA). Details are given in Additional file 1: P4 and P5.

### Quantitative polymerase chain reaction (qPCR)

Quantitative PCR analysis was done to compare the expression levels of listed genes (Primer list is given in the Additional file 1: P6) in control and rd1 eye samples. The relative expression level of each gene was determined by using ΔΔCt method with respect to control samples and normalized with GAPDH levels.

### Analysis of ocular antigens and cyto/chemokines by ELISA

ELISA of mouse TGF-Β1, VEGF, VIP, MIF, IL1RA, S antigen and IRBP (Elabscience®, USA) was performed as per product instruction provided. Serum was used to test free circulating S antigen and IRBP antigens, while ocular lysate was used for TGF-Β1, VEGF, VIP, MIF and IL1RA as given in Additional file 1: P7.

### Western blot

A total of 25 μg protein of both control and test samples were mixed in sample buffer and resolved by 10% SDS-PAGE. Following SDS-PAGE, the proteins were transferred onto 0.20 μm polyvinylidene difluoride (PVDF) membranes and the membranes were blocked with 5% (w/v) non-fat dry milk in TBST for 2 h. After blocking the blots were incubated at 4˚C for overnight with primary antibodies followed by incubation in HRP conjugated secondary antibodies and detection by ECL. GAPDH was then detected post stripping the membrane. Image acquisition was done on Syngene G-box instrument with Syngene software (Syngene, USA). Densitometry of the band images was performed using ImageJ. The band intensity was read and plotted as area under the curve (AUC). Detailed method is given in Additional file 1: section P8.

### Fluorescein angiography

The animal was dark adapted for 30 min and then anesthetized using Ketamine (1 mg/10gm)—xylazine (0.1 mg/10gm). The pupils were dilated using 1% tropicamide, BRB breakdown was studied by intraperitoneal injection of 500ul of 0.2% Sodium Fluorescein dye and imaged in MICRONIII. For quantification of extravasation, and the aqueous humour was isolated 2 min after the IP injection and the absorbance was read at 540 nm. The value was then extrapolated against a calibration curve. Sample preparation is given in Additional file 1: P3.

### Spleen mononuclear cells isolation

Spleen mononuclear cells were isolated from animals by adhesion method. The animal was euthanized and its spleen was isolated. The PBMCs was cells were isolated and 10,000 cells per well was plated in a 24 well plate with RPMI and 2% antibiotic. PBMCs were left unstimulated in a well and the subsequent wells were stimulated with PMA/I (500 ng/50 ng/ml), (20 μg/ml) mouse PBMC lysate and whole retinal lysate (20 μg/ml) for 24 h as given in Additional file 1: P9.

### Immunocytochemistry staining and tissue processing for cryosectioning

Conventional protocols were followed for immunocytochemistry staining of cells for flow cytometry, tissue processing for cryosectioning and immune histochemistry. Detailed procedures are given in Additional file 1: P10, P11 and P12 respectively. Flow cytometry samples were run in Facsverse, the gating strategies are shown in Additional file 1: Fig. S6 and Fig. S7. A representative plot is shown in Additional file 1: Fig. S5. The data was analysed in FlowJo software. Fluorescence microscopy was performed for immune histochemistry in zeiss Imager M2 microscope and analysed in ImageJ.

### ICAM inhibition assay

Intraperitoneal injection of 1 mg/kg of anti-mouse ICAM1 (Immunotools, Germany) antibody in sterile PBS was administered thrice a day for one week in NOD SCID-rd1 mouse. Post 7 days, CFSE labelled 1 million human PBMCs were administered intraperitoneally. The cells were allowed to circulate for 2 h after which the mouse was sacrificed. The eyes were enucleated and a single cell suspension of the retina was obtained as given in Additional file 1: P3. The cells were stained for immune markers and analysed by flow cytometry.

### Statistical analysis

The data was analysed and plotted in GraphPad Prism 5 software. Statistical analysis of difference in normally distributed animal data was performed using parametric t test (Mann–whitney). RP patient data was analysed using non-parametric Krushal-wallis one-way anova with benjamini-hockberg and Wilcox t test. (Prism 5, GraphPad software Inc.) *P* < 0.05 was considered significant. *p* < 0.05 (* significant), *p* < 0.01(**), *p* < 0.001(***) and *P* < 0.0001(****) and *p* values were included in the data.

## Supplementary Information

**Additional file 1.** Additional details of Methods and Results.

## Data Availability

All data generated or analysed during this study are included in this published article [and its supplementary information files].

## Abbreviations

ACAID: Anterior chamber associated immune deviation
AdPs: Adhesion proteins
BRB: Blood retinal barrier
CBA: Cytokine bead assay
COX2: Cyclooygenase
CPD: Citrate phosphate dextrose
CTLA: Cytotoxic T cell associated antigen
ERG: Electroretinography
ICAM: Intracellular adhesion molecule
IHC: Immunohistochemistry
IL1RA: IL1 receptor antagonist
iNOS: Induced nitric oxide synthase
IRBP: Retinol binding protein 3
MCP1: Monocyte chemotactic protein
MIF: Macrophage migration inhibitory factor
OCT: Optimum cutting temperature
PBMCs: Peripheral blood mononuclear cells
PBS: Phosphate saline buffer
Pde6B: CGMP β subunit phosphodiesterase mutation
PDL: Programmed death ligand
PFA: Paraformaldehyde
PMA/I: PMA/inomycin
rd1: Retinal degeneration model 1
RP: Retinitis pigmentosa
RPE: Retinal pigment epithelium
RT: Room temperature
S antigen: Visual arrestin
TGFβ1: Transforming growth factor 1
TJPs: Tight junction proteins
TNFα: Tumor necrosis factor alpha
VCAM: Vascular cell adhesion molecule
VEGF: Vascular endothelial growth factor
VIP: Vasoactive intestinal peptide
ZO: Zona occludin
MFI: Mean fluorescent Intensity
