# Supplementary material for: Immunological consequences of compromised ocular immune privilege accelerate retinal degeneration in retinitis pigmentosa
Source: Orphanet J Rare Dis. 2022 Oct 17;17:378. doi: 10.1186/s13023-022-02528-x (PMC9575261; doi:10.1186/s13023-022-02528-x)
Supplement: Supplementary file 1 — Additional file 1. Additional details of Methods and Results. [file 13023_2022_2528_MOESM1_ESM.pdf]

Supplementary Details

Immunological consequences of compromised ocular immune privilege accelerate retinal degeneration in Retinitis Pigmentosa

Contents

Protocol details

P1. Isolation of Peripheral Blood Mononuclear Cells (PBMCs) page 2

P2. Cytokine Bead Assay page 2

P3. Enucleation page 2

P4. RNA isolation by TRIZOL method page 3

P5. cDNA synthesis page 3

P6. List of used primer page 4

P7. ELISA page 5

P8. Western blotting page 5

P9. Spleen isolation page 5

P10. Flow cytometry staining page 6

P11. Tissue cryo-sectioning page 6

P12. Immunohistochemistry staining page 6

Additional data

S1. Secondary inclusion parameters page 7

S2. Ocular milieu protein profile page 8

S3. Fluorescein Angiography of NOD SCID-rd1 page 9

S4. Fluorescence microscopy of tight junction proteins page 9

S5. Flow cytometry of tight junction proteins page 10

ST1. RP patient clinical information page 11

S6. Flow cytometry gating strategy for retinal cells page 14

S7. Flow cytometry strategy for peripheral immune cells. page 14

Protocol details

P1. Isolation of Peripheral Blood Mononuclear Cells (PBMCs) from buffy coat fraction

PBMCs were isolated from buffy coat fraction by density gradient centrifugation. PBMCs fraction of blood is primarily made up of monocytes and lymphocytes.

1. As buffy coats fraction was diluted with 4 times using incomplete RPMI-1640 media.
2. Diluted blood was carefully layered onto the Hisep™ solution taken into a Falcon tube (Corning, USA) in the ratio of 2:1 without intermixing of two layers.
3. The tubes were then centrifuged at 1000 g for 50 minutes at RT without brake in a swing bucket rotor.
4. Three layers were obtained after centrifugation. The top layer consisting of blood plasma and media used in blood dilution. The second layer is made up of PBMCs and is called as buffy layer, the third layer is Hisep™ underneath which RBCs pellet is obtained.
5. The top layer was discarded carefully by aspiration. Buffy layer was collected carefully avoiding any contamination from third layer.
6. The isolated PBMCs were washed twice using DPBS (Himedia, India) at 300 g for 10 minutes at RT.
7. The PBMCs pellet obtained after washing was re-suspended in 2 ml IMDM media (Gibco).
8. Approximate number of cells isolated was calculated using haemocytometer chamber (Marienfield, Germany) using trypan blue (Sigma) exclusion method.

P2. Cytokine Bead Assay

Ocular lysate was prepared by homogenizing whole eye in sterile PBS followed by centrifuging at 300g 10 mins. The supernatant was used for estimation. 3µl of the requisite cytokine beads (BD biosciences, USA) were added to 15µl of sample and incubated in dark at RT for 3 hours. The samples were washed with wash buffer at 300g for 10 mins. The pellet was then resuspended in 200µl wash buffer and the presence of respective cytokines was detected by flow cytometry (FACSVerse).

P3. Enucleation

Whole eye lysate was used for ocular milieu studies and the sections prepared from posterior eyecup was used for immunohistochemistry staining. RPE single cell suspension was obtained by mechanical disruption of the layer. The suspension was filtered through a 70-micron nylon filter and was used for flow cytometry studies. Enucleated eyeballs were micro-dissected to obtain the posterior cup containing the neural retina layer and the RPE layer, which was used for further analysis. The enucleated the eyeball in the socket with the third eyelid by carefully and slowly cutting through the extraocular muscles and severing the optic nerve. The eyeball

was pierced through the corneal-limbus using a microblade. The incision was used as the point of entry thereby cutting along the corneal- scleral junction by inserting micro-scissors through the incision point. Small and continuous cuts along the circumference were made till the cups separated. They can be further dissected by gently peeling the translucent layer of the neural retina using Colibri suturing forceps thus, obtaining neural and RPE layer. Further, three equidistant cuts are made from the periphery perpendicularly to the optic centre and till the optic nerve. This opens the hemispherical cups into a flower shape which falls flat and can be easily mounted. Whole eye lysate was used for ocular milieu studies and the sections prepared from posterior eyecup was used for IHC. Mechanical disruption was used to obtain RPE single cell suspension. The suspension was filtered through a 70-micron nylon filter and was used for flow cytometry studies. Aqua-centesis was performed to draw aqueous humour via trans-corneal injection. 5µl sterile PBS was injected and re-drawn to acquire aqueous flush.

P4. RNA isolation by TRIZOL method.

TRIZOL or guanidium thiocynate-phenol-chloroform method was used to isolate RNA from tissue.

1. Chloroform was added to the TRIZOL (Takara) suspension of ground tissue with the ratio of 200 µl chloroform added to per ml of TRIZOL reagent.
2. The two reagents were mixed by inverting the tube 10-15 times.
3. Tube was incubated at RT for 10 minutes.
4. The tube was then centrifuged at 12000 g for 30 minutes at 4˚C.
5. After centrifugation the reagents separated into two phases. Upper aqueous phase containing RNA and lower organic phase containing DNA. The phases were separated by a white interphase which consists of all protein component. The aqueous phase was collected for further processing.
6. Isopropanol was added to the aqueous phase with the ratio of 500 µl isopropanol added to per ml of TRIZOL reagent.
7. The tube was inverted for 15-20 times for proper mixing followed by incubation at RT (24˚C) for 15 minutes.
8. The tube was then centrifuged at 12000 g for 40 minutes at 4˚C.
9. After centrifugation supernatant was removed and RNA pellet was washed two times with 1.0 ml of 75% ethanol prepared in nuclease free molecular grade water.
10. After washing the RNA pellet was dried until the pellet had turned transparent.
11. The pellet was then dissolved in 20-50 µl of nuclease free molecular grade water.

P5. cDNA Synthesis

The reaction was carried out in a thermal cycler as follows:

Component Volume
5X iScript Buffer 4 µl
RT Enzyme 1 µl
Nuclease free molecular grade MQ X µl

Total Reaction volume 20 µl
RNA Template (1µg total RNA)
Cycle
25 ˚C 5 minutes
46 ˚C 20 minutes
95 ˚C 4 minutes

Hold

P6. List of used primer

No.	Gene	Forward	Reverse
1.	iNOS	GCAAGAGAGTGCTGTTCC	TGTCCTGAACGTAGACCTT
2.	COX2	TCTCCAACCTCTCCTACTACA	CTCCTTATTTCCCTTCACACCC
3.	CateninA	TACAGAACCTTGGTGGAGA	CACAACAGCATTCATCAAGTTC
4.	CateninD	CATCCGCTCTGCTCTTCGT	ACTACTCGCTCATGCTCACTC
5.	CateninB	GATGATCCCAGCTACCGTTCTTT	TGCTCCATCATAGGGTCCATCC
6.	E cadherin	AAGACCAGGACTTTGATTTGAGC	GCCACATCATTTCGAGTCACT
7.	N cadherin	AGAAGACCAGGACTATGACTTGAG	GTCTGATTCCCACGGGCTT
8.	ZO3	CTTCACCGCCACCATCCC	GCCTGCTGTTGCTGTATT
9.	ZO2	AGACCAACTTAGGGATGCTA	CTTCTCTGTTCTGGCTTCTTG
10	ZO1	CGTAGTGCTCAGAGGTAA	CAGAATACGGCTCCTTCCT
11	jamA	AAGGTCATTTACAGCCAGCCCAG	CAGCAGGTCACACCAGGAAC
12	jam3	TGCGTACAGACGAGGCTGCTT	CCGTCATGCTTCCCTGGGCTC
13	claudin5	GCGAACAGTTCCTACTGAGATCCT	CCTTTCAGGTTAGCAGGTGCC
14	Occludin	CTGCTGCTGATGAATATAATAG	CCTCTTGATGTGCGATAA
15	Cingulin	TGGAGGAACAGCATGAGGCTAATG	AGCTTCCGCCAGGCATCC
16	Afadin	ACAAGGTGGACCGTACTG	TCCTGTAGTCTCTTCTGGAAC
17	ZONAB	AACGCTGTTTCACAAGATGGCA	GCACTGCTCTGTTCGGTAG
18	Pard3	CTTTGAGCAATCTTGGTCC	GTACTTGAACCTCCACGG
19	Symplekin	CATCCAGTCCTCACCAT	GTACTTCCATACCTGCTTCAT

P7. ELISA

Blood was collected from animals by retro orbital bleeding. For serum collection, blood was collected in 1.5 ml Eppendorf tubes without the addition of any anti-coagulant. For the purpose of cell staining, blood was collected in Citrate Phosphate Dextrose (CPD) solution containing tubes. Serum was isolated from blood for ELISA. Blood collected in a tube without any anticoagulant was kept at RT to clot for 30 minutes, after which the tube was centrifuged at 10000g at RT for 20 minutes. Serum was collected and stored at -80˚C.

100µl of sample and standard was added to the precoated wells for 90 minutes. They were then incubated with biotinylated detection antibody for 60 minutes followed by three washes. HRP conjugate was then added to each well and incubated for 30 minutes followed by 5 washes, following which the substrate reagent was added and incubated for 15 minutes. After colour development, stop solution was added and the plate was read at 450nm immediately.

P8. Western Blot

Expression level of selected differentially expressed proteins (DEP) was further validated using Western blotting. A total of 25-30 µg protein of both control and test samples were mixed in sample buffer (50 mM Tris, pH 6.8, 10% glycerol, 2% SDS and 0.01% bromophenol blue) and resolved by 10% SDS-PAGE. Following SDS-PAGE, the proteins were transferred onto 0.20µm polyvinylidene difluoride (PVDF) membranes (Pall Life Sciences, Port Washington, NY, USA). The membranes were blocked with 5% (w/v) non-fat dry milk in TBST [50 mM Tris, pH 8.0, 150 mM NaCl, and 0.1% Tween-20 (v/v)] for 2 h. After blocking the blots were incubated at 4˚C for overnight with primary antibodies (dilution 1:1000, VEGF Affinity biosciences Rabbit mAb AF5131) followed by incubation in HRP conjugated secondary antibodies and detection by ECL (Bio Rad Labs, USA). The membrane was then stripped using stripping buffer for 30 minutes and incubated with GAPDH (dilution 1:1000, sc-47724, Santa cruz) followed by incubation in HRP conjugated secondary antibodies and detection by ECL (Bio Rad Labs, USA). Image acquisition was done on Syngene G-box instrument with Syngene software (Syngene, USA). Densitometry of the band images was performed using ImageJ. The band intensity was read and plotted as area under the curve (AUC).

P9. Spleen mononuclear cells isolation

Spleen mononuclear cells were isolated from animals by adhesion method. The animal was euthanized and its spleen was isolated. The spleen was crushed between two frosted slides and washed with RPMI. The cells were then filtered through 70micron filter, followed by centrifuge at 300g for 10 mins. The pellet was resuspended and plated in T25 flask and left overnight. The supernatant containing mononuclear cells was collected and washed with DPBS at 300g for 10 mins. The cells were counted and 10,000 cells per well was plated in a 24 well plate with RPMI and 2% antibiotic. PBMCs were left unstimulated in a well and the subsequent wells were stimulated with PMA/I (500ng/50ng/ml), (20µg/ml) mouse PBMC lysate and whole retinal lysate(20µg/ml) for 24 hrs. The cells were then centrifuged at 300g for 10 minutes. The supernatant was collected and stored and the cell pellet was fixed with 4% PFA for further analysis.

P10. Immunocytochemistry staining of cells for Flow Cytometry

In the current study, flow cytometry was done to determine the immune marker positive cells in blood and tissue cellular suspension. The cells were incubated in 1:200 dilution of respective fluorochrome conjugated antibodies for 40 mins at 4ºC. The cells were then washed in PBS by centrifuging at 300g for 10 minutes. The cell pellet obtained was resuspended in 200µl PBS and run in BD FACs Verse flow cytometer.

Flow cytometry was done to determine the protein expression on retinal cells suspension. The cells where incubated in 1:200 dilution of respective fluorochrome conjugated antibodies for 40 minutes at 4ºC with TGFB1 tagged with PE. The cells were the washed with and PBS and pellet obtained was resuspended in 200µl PBS and run in BD FACs Verse flow cytometer. To detect ZO1 and E cadherin, retinal single cell suspension was incubated with untagged ZO1 and E cadherin (affinity biosciences, AF5145 and AF0131) primary antibody at 1:100 dilution. The cells were then washed in PBS by centrifuging at 300g for 10 minutes. The untagged cells were incubated with secondary anti-rabbit Alexa 488 flour antibody for 40 minutes. The cells were the washed with and PBS and pellet obtained was resuspended in 200µl PBS and run in BD FACs Verse flow cytometer. The mean fluorescence intensity (MFI) was read to determine the protein expression profile.

P11. Tissue processing for cryosectioning.

Tissue samples were fixed in 4% Para-formaldehyde (PFA) solution for 4 hrs at 4˚C followed by dehydration of tissue for at least 24 hrs in 30% sucrose solution in PBS. For block making tissue samples were embedded in Optimal Cutting Temperature (OCT) compound supplied by Tissue-Tek, Sakura Finetek, USA. The OCT blocks were cut using Shandon Chryotome E (Thermo Electron Corporation, USA) into sections of thickness of 4 µm and mounted on poly-L-lysine coated glass slides.

P12. Immune histochemistry staining

The tissue sections obtained on poly-L-lysine coated slides. Thereafter, the cells/tissue were fixed with 4% PFA for at least 30 minutes at 4oC in dark. The fixed cells/tissue were permeabilized for 45 minutes using permeabilization buffer containing saponin, followed by washing and blocking with 5% bovine serum albumin (BSA) for 45 minutes at RT. Post blocking, the cells/tissue were washed with wash buffer and incubated in primary antibodies of appropriate dilutions in permeabilization buffer for at least an hour at RT or overnight at 4oC. The cells/tissue were then washed thrice with wash buffer, each for 10 minutes, and then incubated with a fluorochrome labeled secondary antibody for 40 - 45 minutes at RT followed again by three washes. Thereafter, the cells/tissue were counterstained with 4', 6-Diamidino-2-Phenylindole dihydrochloride (DAPI) (dilution 1:500; stock concentration: 1 mg/ml) for 5 minutes at RT. The DAPI stained cells/tissue were further washed with PBS and mounted with coverslips using either 70% glycerol solution or permanent mounting media Vectashield (ThermoScientific, USA). Representative images were captured using a fluorescent microscope.

Additional data

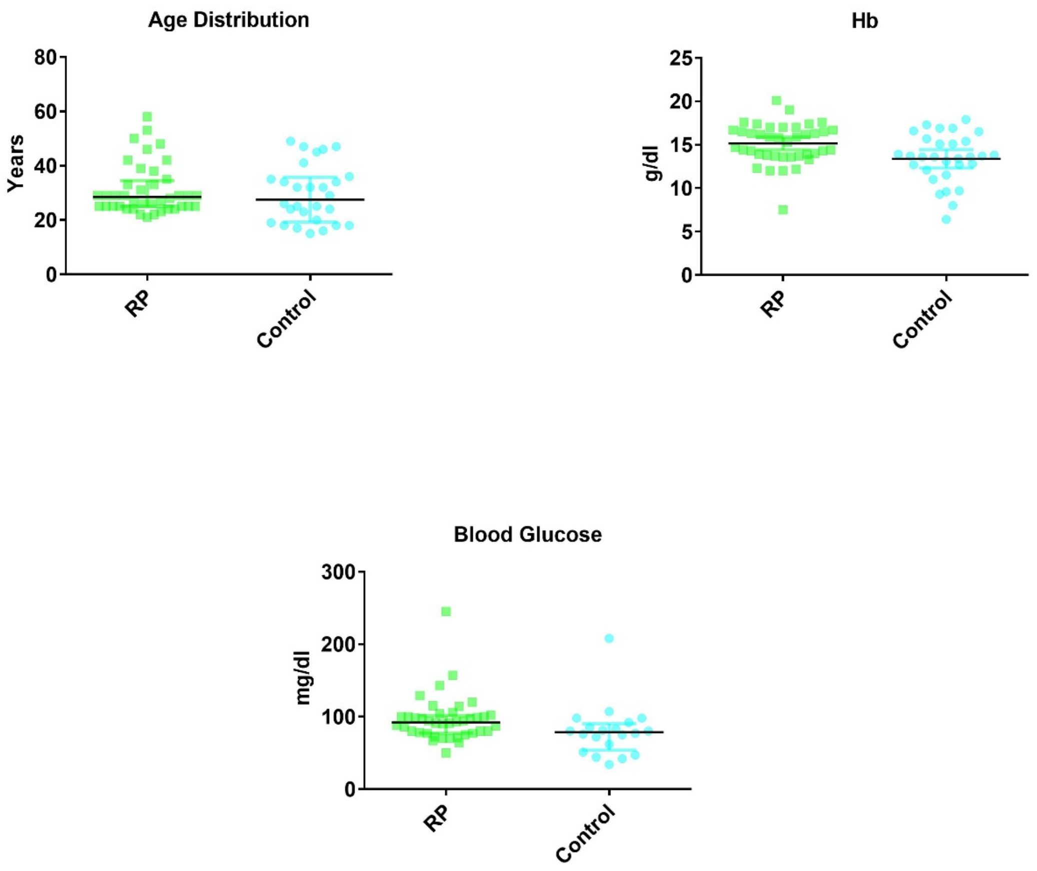

Fig. S1. Secondary inclusion parameters. RP patients between the age of 18 to 60 were recruited with normal health parameters assessed by haemoglobin levels and glucose levels. The inclusion parameters eliminated nutritional deficiency and uncontrolled blood glucose levels mediated ocular defects which can alter vision perception and blood parameters. Age matched controls with normal health factors were recruited. Healthy n= 30 RP=40.

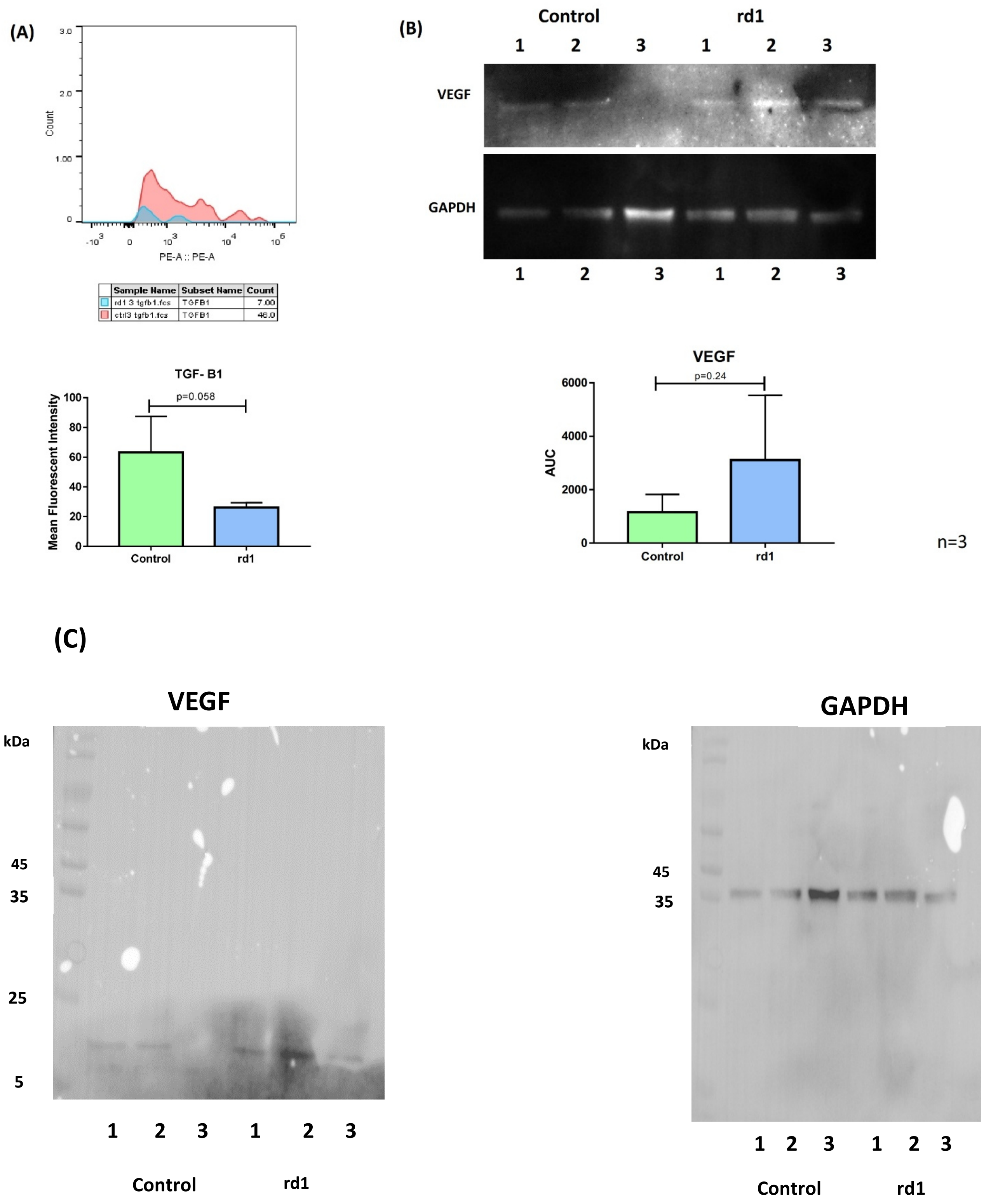

Fig. S2. Ocular milieu. Flow cytometry indicated that TGF-B1 was downregulated in rd1 (p= 0.058) (A), the western blot of VEGF confirmed its overexpression in rd1 eyes compared to C57BL/B6 (B) n=3. The complete blot images of VEGF at 16kDa and internal control GAPDH at 37KDa (on the same blot post stripping) has been indicated with the molecular marker (C).

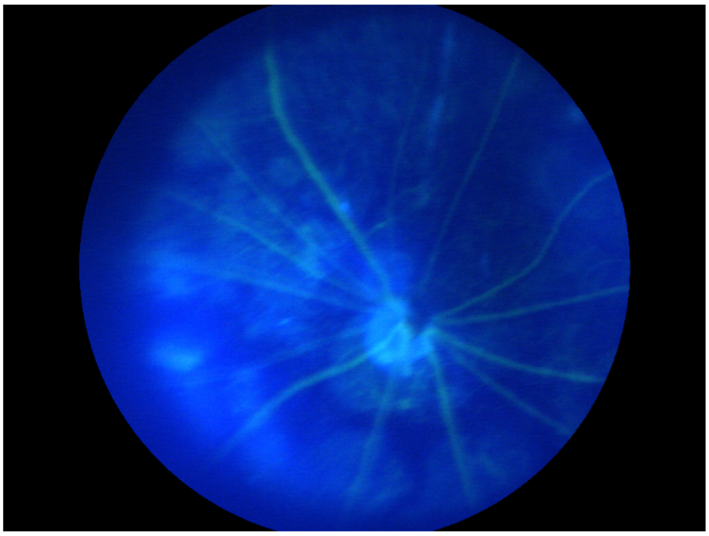

Fig. S3. Fluorescein Angiography on NOD SCID-rd1 at P28. The fluorescein dye extravasated from retinal vessels in to retinal space. However, not to the degree seen in rd1. Thus, the BRB was compromised in NOD SCID-rd1 due to ocular inflammation. The breakdown was more pronounced in rd1.

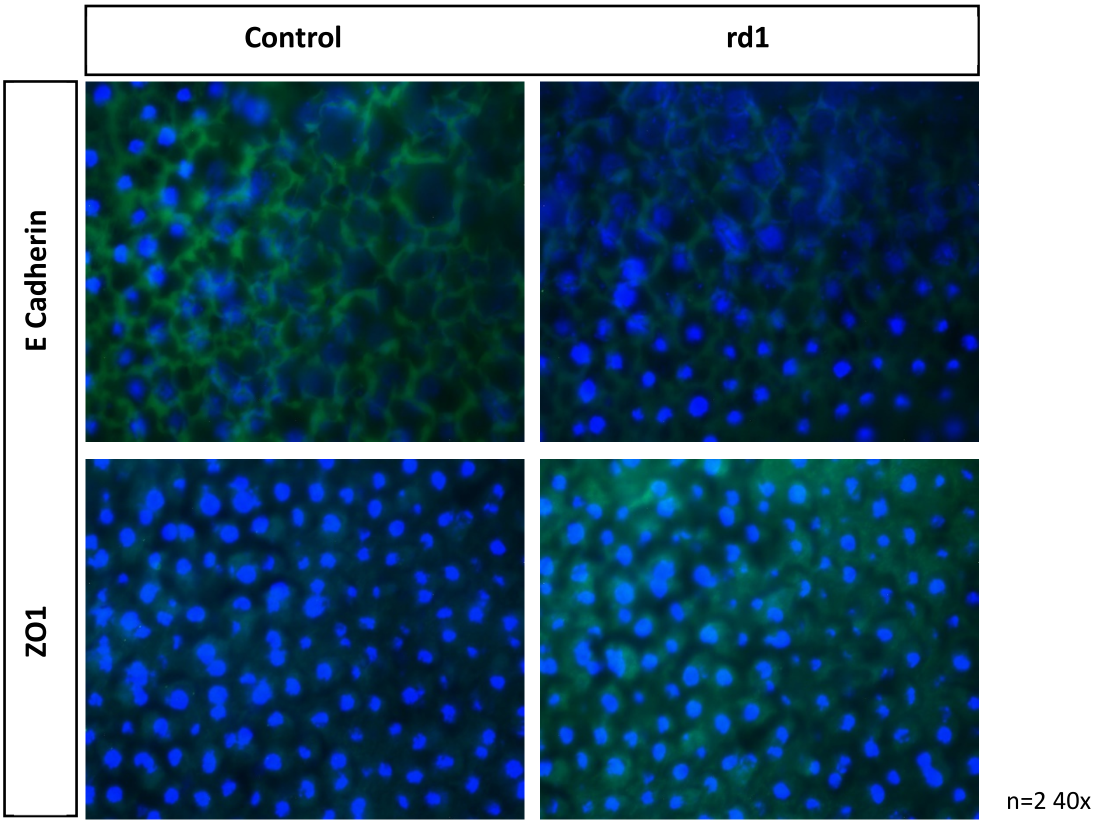

Fig. S4. Fluorescence microscopy of tight junction proteins. E cadherin TJP was found to be downregulated in rd1 (A), while ZO1 was found to be overexpressed in rd1 RPE cells compared to C57BL/B6 (B). Green: Alexa 488: ZO1/E cadherin. Blue: DAPI

(A)

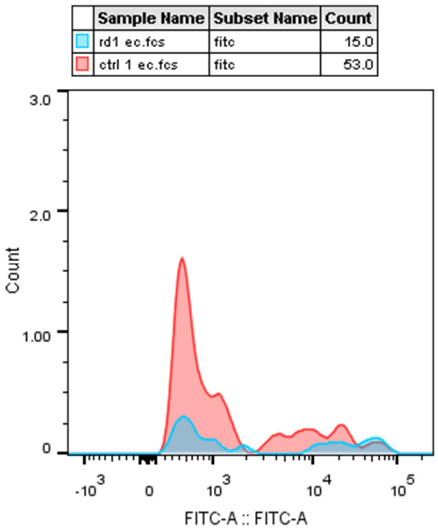

(B)

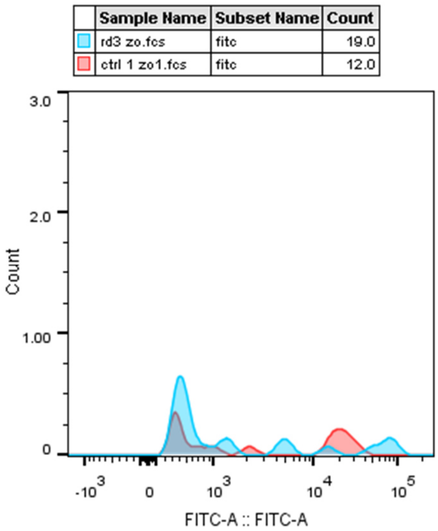

Fig. S5. Flow cytometry of tight junction proteins. Representative flow cytometry histogram overlay plots of E cadherin (A) and ZO1 (B). E cadherin was found to be significantly downregulated in rd1, while ZO1 was found to be overexpressed in rd1 RPE cells compared to C57BL/B6.

A few details of RP patients

S.no.	Age of onset of symptoms	Mode (acute vs gradual)	Inheritance mode (pedigree charting)	Phenotypic details	Remarks
1	Unknown	Gradual	Autosomal Recessive	Waxy pallor, bony spicules, Night blindness, tunnel vision	Ceramide kinase mutation
2	10 years	Gradual	Autosomal recessive	Waxy pallor, bony spicules, Night blindness, tunnel vision, photo-sensitivity.	
3	15 years	Gradual	Autosomal recessive	Waxy pallor, bony spicules, Night blindness, tunnel vision, photo-sensitivity.	
4	36 years	Gradual	Autosomal recessive	Waxy pallor, bony spicules, Night blindness, tunnel vision, photo-sensitivity.	Consanguinity
5	15 years	Gradual	Autosomal recessive	Waxy pallor, bony spicules, Night blindness, tunnel vision, photo-sensitivity.	
6	17 years	Gradual	Autosomal recessive	Waxy pallor, bony spicules, Night blindness, tunnel vision, photo-sensitivity.	
7	Not known	Acute	Autosomal recessive	Waxy pallor, bony spicules, Night blindness, tunnel vision, photo-sensitivity.	RPE65 mutation
8	20 years	Gradual	Autosomal recessive	Waxy pallor, bony spicules, Night blindness, tunnel vision, photo-sensitivity.	Consanguinity
9	17 years	Gradual	Not known	Waxy pallor, bony spicules, Night blindness, tunnel vision, photo-sensitivity.	
10	27 years	Gradual	Not known	Waxy pallor, bony spicules, Night blindness, tunnel vision, photo-sensitivity.	
11	20 years	Gradual	Autosomal recessive	Waxy pallor, bony spicules, Night blindness, tunnel vision, photo-sensitivity.	Consanguinity
12	15 years	Acute	Not known	Waxy pallor, bony spicules, Night blindness, tunnel vision, photo-sensitivity.	
13	16 years	Gradual	Autosomal recessive	Waxy pallor, bony spicules, Night blindness, tunnel vision, photo-sensitivity.	
14	22 years	Gradual	Not known	Waxy pallor, bony spicules, Night blindness, tunnel vision, photo-sensitivity.	
15	15 years	Gradual	Autosomal Recessive	Waxy pallor, bony spicules, Night blindness, tunnel vision, photo-sensitivity.	Consanguinity
16	Unknown	Acute	Autosomal Recessive	Waxy pallor, bony spicules, low vision and hearing loss	Usher's Syndrome

17	11 years	Acute	Not known	Waxy pallor, bony spicules, Night blindness, tunnel vision, photo-sensitivity.	
18	22 years	Gradual	Not known	Waxy pallor, bony spicules, Night blindness, tunnel vision, photo-sensitivity.	
19	10 years	Gradual	Autosomal recessive	Waxy pallor, bony spicules, Night blindness, tunnel vision, photo-sensitivity.	Consanguinity
20	15 years	Gradual	Not known	Waxy pallor, bony spicules, Night blindness, tunnel vision, photo-sensitivity.	
21	16 years	Gradual	Autosomal Recessive	Waxy pallor, bony spicules, Night blindness, tunnel vision, photo-sensitivity.	Consanguinity
22	21 years	Gradual	Autosomal Recessive	Waxy pallor, bony spicules, Night blindness, tunnel vision, photo-sensitivity.	Consanguinity
23	18 years	Gradual	Autosomal Recessive	Waxy pallor, bony spicules, Night blindness, tunnel vision, photo-sensitivity.	Consanguinity
24	19 years	Gradual	Not known	Waxy pallor, bony spicules, Night blindness, tunnel vision, photo-sensitivity.	
25	20 years	Gradual	Autosomal Recessive	Waxy pallor, bony spicules, Night blindness, tunnel vision, photo-sensitivity.	Consanguinity
26	16 years	Gradual	Not known	Waxy pallor, bony spicules, Night blindness, tunnel vision, photo-sensitivity.	
27	21 years	Gradual	Autosomal Recessive	Waxy pallor, bony spicules, Night blindness, tunnel vision, photo-sensitivity.	Consanguinity
28	Childhood onset (age Not known)	Gradual	Autosomal Recessive	Waxy pallor, bony spicules, Night blindness, tunnel vision, photo-sensitivity.	Consanguinity
29	22 years	Acute	Autosomal Recessive	Waxy pallor, bony spicules, Night blindness, tunnel vision, photo-sensitivity.	Consanguinity
30	13 years	Gradual	Not known	Waxy pallor, bony spicules, Night blindness, tunnel vision, photo-sensitivity.	
31	22 years	Acute	Autosomal Recessive	Waxy pallor, bony spicules, Night blindness, tunnel vision, photo-sensitivity.	
32	23 years	Gradual	Autosomal Recessive	Waxy pallor, bony spicules, Night blindness, tunnel vision, photo-sensitivity.	Consanguinity
33	24 years	Gradual	Autosomal Recessive	Waxy pallor, bony spicules, Night blindness, tunnel vision, photo-sensitivity.	Consanguinity
34	22 years	Gradual	Not known	Waxy pallor, bony spicules, Night blindness, tunnel vision, photo-sensitivity.	

35	44 years	Gradual	Autosomal Recessive	Waxy pallor, bony spicules, Night blindness, tunnel vision, photo-sensitivity.	
36	11 years	Acute	Not known	Waxy pallor, bony spicules, Night blindness, tunnel vision, photo-sensitivity.	
37	26 years	Gradual	Autosomal Recessive	Waxy pallor, bony spicules, Night blindness, tunnel vision, photo-sensitivity.	Consanguinity
38	23 years	Gradual	Not known	Waxy pallor, bony spicules, Night blindness, tunnel vision, photo-sensitivity.	
39	22 years	Gradual	Autosomal recessive	Waxy pallor, bony spicules, Night blindness, tunnel vision, photo-sensitivity.	
40	12 years	Acute	Autosomal Recessive	Waxy pallor, bony spicules, Night blindness, tunnel vision, photo-sensitivity.	

Table ST1. Information about type of RP, age of onset of symptoms and mode (acute vs gradual) and inheritance mode and phenotypic details used to formulate the diagnosis has been provided in the table to allow confirmation of the diagnostic conclusion.

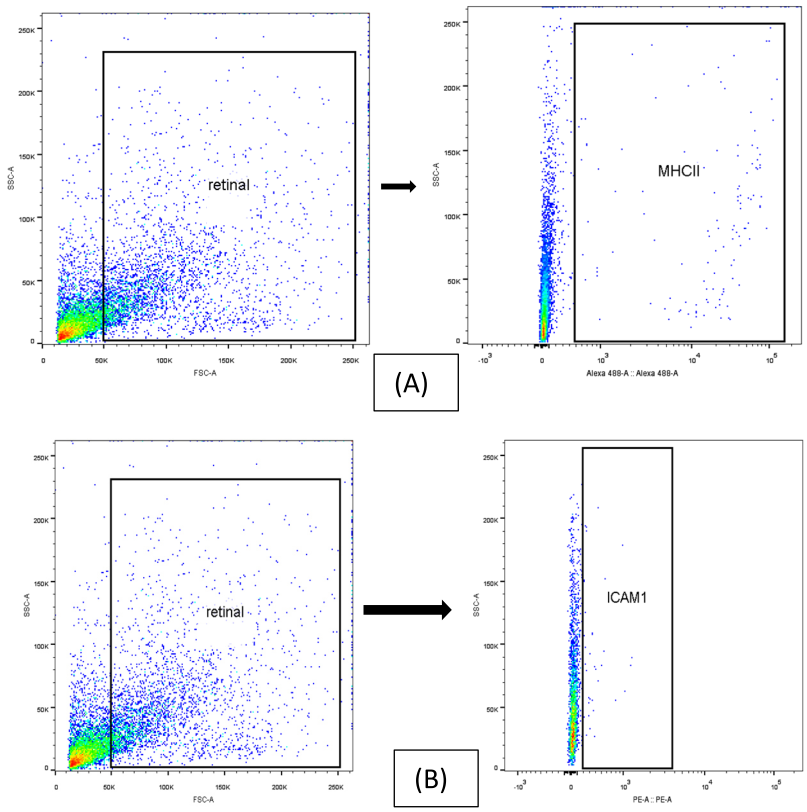

Fig. S6. The gating strategy used in flow cytometry. Primary gate P1 was applies on retinal neuronal cells leaving the debris. P1 was applied for FITC positive MHCII (A) ICAM1(B). The same gating strategy was applied for retinal cells study.

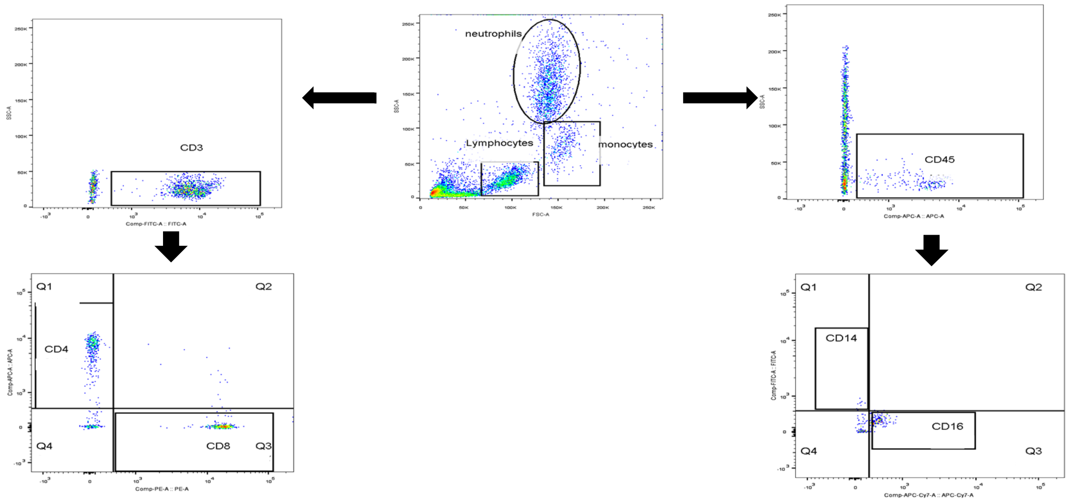

Fig. S7. The gating strategy used in flow cytometry for peripheral immune cells. The FSC and SSC were modulated to attain a scatter plot with lymphocytes, monocytes and granulocytes. The clusters were gated for sub-gating. T cell were gating for CD3(FITC) positive cells and subsequently gated for CD4+(APC) and CD8+ (PE). The monocytes were gated for CD45+ (APC) and sub-gated for CD14+ (PE) and CD16+ (FITC).
